# Épidémiologie descriptive de la carcinose péritonéale d’origine digestive à l’Hôpital Universitaire Ibn Rochd de Casablanca (2008-2010)

**DOI:** 10.11604/pamj.2017.27.234.13276

**Published:** 2017-07-31

**Authors:** Mohammed Benlahfid, Driss Erguibi, Khalid Elhattabi, Fatimazahra Bensardi, Driss Khaiz, Rachid Lafriekh, Dounia Rebroub, Abdelaziz Fadil, Touria Aboussaouira

**Affiliations:** 1 Université Hassan II, Faculté de Médecine et de Pharmacie, CEDoc, Casablanca, Maroc; 2 Université Hassan II, Faculté de Médecine et de Pharmacie, Casablanca, Service de Chirurgie Générale, CHU Ibn Rochd, Casablanca, Maroc; 3 Université Hassan II, Faculté de Médecine et de Pharmacie, Casablanca, Service des Urgences Chirurgicales, CHU Ibn Rochd, Casablanca, Maroc

**Keywords:** Epidémiologie, carcinose péritonéale, digestif, Epidemiology, peritoneal carcinosis, digestive

## Abstract

**Introduction:**

La carcinose péritonéale est une diffusion inéluctablement terminale chez les patients atteints de cancers abdominaux. C'est le signe d'une maladie avancée ou d'une ré-évolution le plus souvent associée à un pronostic sombre. Environ deux tiers de l'ensemble des carcinoses péritonéales sont d'origine digestive et un tiers d'origine non digestive.

**Méthodes:**

Il s'agit d'une étude rétrospective descriptive menée entre janvier 2008 et décembre 2010, dans le but de dresser le profil épidémiologique et les facteurs de risques de la carcinose péritonéale d'origine digestive au Centre Hospitalier Universitaire de Casablanca.

**Résultats:**

Quarante-sept cas de carcinose péritonéale d'origine digestive ont été recensées (22 femmes, 25 hommes) ce qui représente une prévalence de 6.19% et un nombre moyen de 15.6 cas par an. L'âge était le facteur de risque essentiel dans notre série avec un âge moyen de 55.55 ans ±12.32. Les antécédents familiaux présentaient aussi un facteur de risque à prendre en considération.

**Conclusion:**

A travers notre étude, nous avons conclus que les principaux facteurs de risque de la carcinose péritonéale d'origine digestive au Centre Hospitalier Universitaire Ibn Rochd Casablanca, sont l'âge et les antécédents familiaux.

## Abstract (en)

**Introduction:**

Peritoneal carcinosis is characterized by ineluctably terminal diffuse spread of abdominal cancer. It is the sign of an advanced disease or a re-emerging disease most often associated with a dark prognosis. Approximately two thirds of all peritoneal carcinomas are of gastrointestinal origin and one third are of non-digestive origin.

**Methods:**

We conducted a retrospective descriptive study between January 2008 and December 2010 in order to establish epidemiological features and risk factors of peritoneal carcinosis of gastrointestinal origin at the Ibn Rochd University hospital, Casablanca.

**Results:**

Forty-seven cases of peritoneal carcinosis of gastrointestinal origin were recorded (22 women, 25 men), corresponding to a prevalence of 6.19% and to a mean number of 15.6 cases per year. Age was the major risk factor in our case series (with an average age of 55.55 ± 12.32 years). Even family history was a risk factor to consider.

**Conclusion:**

This study concluded that the major risk factors for peritoneal carcinosis of digestive origin are age and family history at the Ibn Rochd University hospital, Casablanca (2008-2010).

## Introduction

La carcinose péritonéale (CP) d'origine des cancers digestifs est une diffusion terminale et est le plus souvent associée à un pronostic sombre. Environ deux tiers de l'ensemble des CP sont d'origine digestive et un tiers d'origine non digestive. Parmi ces CP, 40% sont d'origine pancréatique, 30 à 40% d'origine gastrique et 20% sont d'origine colorectale et le reste d'origines diverses [1]. Avec une survie médiane de 12 mois, le pronostic des CP d'origine ovarienne est meilleur que celui des CP d'origine colorectale (survie médiane de 4 à 7 mois), lui-même meilleur que celui des CP d'origine gastrique ou pancréatique (survie médiane de 1 à 3 mois) [1, 2]. La CP représente la lésion la plus fréquente du péritoine, elle peut être synchrone ou métachrone de la découverte du cancer primitif. L'atteinte péritonéale se fait selon quatre voies: par contigüité, par diffusion péritonéale (cas dus cancers digestifs et ovariens), par voie hématogène (mélanome, cancer pulmonaire et du sein) ou par voie lymphatique. Une fois la cavité péritonéale atteinte, les cellules tumorales migrent dans la cavité selon la circulation des fluides [3]. Le cancer gastrique occupe le premier rang des cancers digestif au Maroc. A Rabat, c'est le cancer digestif le plus fréquent. L'âge médian est de 65 ans chez les hommes et 50 ans chez les femmes [4]. A Marrakech Une étude rétrospective sur 440 cancers digestifs admis au service d'oncologie radiothérapie durant la période de 2003 à 2007 a présentée les valeurs suivantes: les cancers digestifs constituent 12.62% de l'ensemble des cancers, le cancer colorectal constituait le cancer le plus fréquent avec 35.68% des cas, suivie du cancer de l'estomac qui occupait la seconde position avec 33.86% des cas [5]. Près de 80% des patients nouvellement diagnostiqués de cancers digestifs, sont déjà au stade métastatique de la maladie, pour laquelle aucun traitement curatif n´est actuellement disponible [6]. Les autres patients qui relèvent d'un traitement palliatif médical ou chirurgical ont une survie médiane qui n'excède pas 6 à 8 mois [7]. L'objectif de cette étude est de dresser, rétrospectivement à partir d'une base de données, le profil épidémiologique des CP d'origine digestive, confirmées histologiquement sur une période de 3 ans.

## Méthodes

Il s'agit d'une étude rétrospective, descriptive étalée sur une période de trois années, entre le premier Janvier 2008 et le 30 Décembre 2010, évaluant la fréquence des CP d'origine digestive au CHU Ibn Rochd de Casablanca. A partir des registres de consultation et des dossiers médicaux nous avons relevé pour chaque malade les paramètres suivants: l'âge, le sexe, les données démographiques, les antécédents et les signes cliniques révélateurs, ces données ont été récupérées sur des fiches d'exploitation, saisies et analysées grâce au logiciel statistique SPSS 16.0. Pour les tests khi^2^ et Anova à un facteur utilisés, le résultat est considéré significatif lorsque p < 0.05. Les variables quantitatives ont été décrites selon la moyenne et l'écart-type ainsi que le minimum et le maximum. Les variables qualitatives ont été décrites selon la fréquence et le pourcentage.

## Résultats

**Fréquence**: Au total, 47 cas de CP (confirmé histologiquement) ont été recensés rétrospectivement au CHU de Casablanca sur la période étalée de 2008 à 2010, soit une prévalence de 6.19% et un nombre moyen de 15.6 cas de CP d'origine digestive par an.

**Figure 1:**
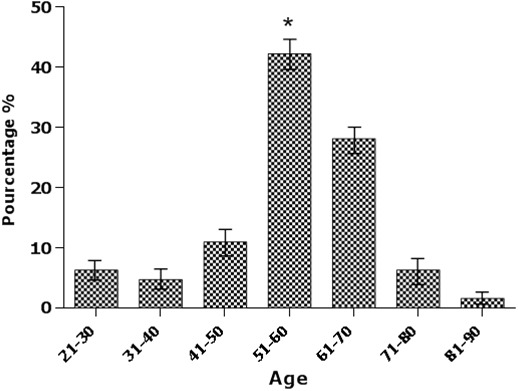
Répartition des malades avec carcinose péritonéale d’origine digestive par tranche d’âge (p < 0.001)

**Figure 2:**
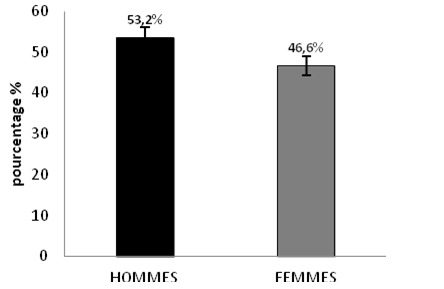
Répartition des patients avec carcinose péritonéale d’origine digestive selon le sexe

**Répartition selon l'âge et le sexe**: Selon nos résultats, la moyenne d'âge au moment du diagnostic des patients était de 55.55 ans ± 12.32, avec des extrêmes à 20 ans et 85 ans. Le risque de survenue de la CP est d'autant plus important que l'on est âgé et la tranche d'âge 51 à 60 ans était la plus touchée avec 42.2% (Figure 1). Selon nos calculs, nos patients se répartissaient en 25 hommes (53.2%), 22 femmes (46.8%) avec une prédominance masculine, soit un Sex-ratio de 0.88 (Figure 2).

**Répartition selon l'origine et les antécédents**: D'après les données statistiques de notre étude, la majorité de nos patients (30 cas soit 63.8%) sont issus du milieu urbain alors que 16 cas (soit 34%) sont issus du milieu rural, à noter qu'un seul patient ne disposait pas de renseignements sur son origine. Nos résultats montrent que le diabète était l'antécédent le plus fréquent dans notre série avec 46.7% alors que les antécédents familiaux du coté paternel n'ont été mentionnés que chez 4.3% des patients (Tableau 1). Les antécédents familiaux du coté fraternel (14.9%) présentaient une différence statiquement significative (p= 0.016).

**Tableau 1:** Les antécédents des patients avec CP d’origine digestive

Les Antécédents	N	Pourcentage %
Diabète	22	46.7
HTA	20	42.6
Chirurgie	16	34
Alcool	8	17.4
Tabac	11	23.4
Cannabis	4	8.5
Familiaux paternel	2	4.3
Familiaux fraternel	7	14.9

**Figure 3:**
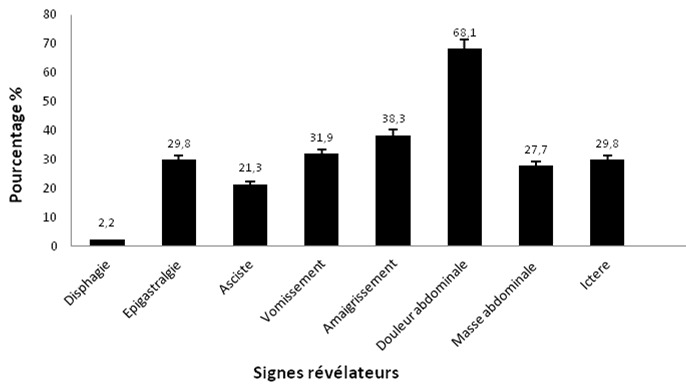
Différents signes révélateurs chez des patients avec carcinose péritonéale d’origine digestive

**Répartition selon les signes cliniques**: La majorité de nos patients (soit 60.75%) ont consulté un médecin dans les 6 mois qui suivait le début des signes cliniques fonctionnels. Par contre une proportion importante de patients (39.25%) n'a eu recours à la médecine moderne qu'après 6 mois, voire 1 an d'évolution. La **Figure 3** rend compte les signes cliniques relevés à l'admission des malades et nous retenons que 2.2% de nos malades se plaignaient de dysphagie alors que ceux qui se plaignaient des douleurs abdominales étaient plus fréquent avec 68.1%.

## Discussion

La CP est une entité hétérogène regroupant des affections dont le pronostic dépend du primitif. Ce pronostic spontané très péjoratif de cette pathologie d'origine digestive (médiane de survie inférieure à 8 mois) a été confirmé par deux études prospectives [1, 8] et par une large étude rétrospective [2]. La survie des patients porteurs d'une CP est difficile à évaluer, elle dépend avant tout du cancer primitif, mais aussi de l'étendue de la CP. L'âge moyen de nos patients atteints de CP est de 55.55 ans avec un âge médian de 56 ans, ceci confirme relativement les données de la littérature [4, 9], cependant 78.13% de nos patients ont un âge de plus de 50 ans ce qui confirme les données de la littérature que cette pathologie évolue insidieusement pendant plusieurs mois voire des années et reste asymptomatique, ou s'exprime par des signes trompeurs et non spécifiques ou également être découverte fortuitement lors d'une intervention chirurgicale. Tandis que seulement 21.87% des patients sont diagnostiqué à un âge jeune. L'analyse statistique de nos résultats a permis de déterminer que le facteur âge était associé de façon significative à la prévalence des patients atteint de CP d'origine digestive (p = 0.001). La prédominance masculine (avec un sex-ratio de 0.88 dans notre série) ainsi que l'atteinte prédominante des personnes âgées de plus de 50 ans rejoignent les données de la littérature [4, 9]. Selon nos statistiques, les antécédents familiaux (fratrie p = 0.016) présentent une différence significative, donc ces antécédents peuvent être considérer comme un facteur de risque important de la CP d'origine digestive.

Trente cas soit 63.8% de notre série sont issus du milieu urbain alors que 16 cas seulement (soit 34%) sont issus du milieu rural. La majorité de nos patients sont issus du milieu urbain étant donné que le Centre Hospitalier Universitaire draine essentiellement la région du grand Casablanca, ainsi que le centre d'oncologie du CHU constitue une structure spécialisée ce qui explique le pourcentage important de 16% aussi des patients du milieu rural. La CP a longtemps été synonyme de stade terminal incurable des cancers digestifs, la quasi-totalité (90%) des patients décède dans les deux ans qui suivent le diagnostic de CP, en France quarante pour cent (40%) des cancers digestifs sont associés ou vont évoluer vers une CP, soit 2800 nouveaux cas par an [10], sa gravité s'explique par la rareté d'un diagnostic précoce et par la rapidité de l'extension locorégionale puis métastatique (au moment du diagnostic, 20 à 30% des malades ont une tumeur localement avancée, 50% ont déjà des métastases) [11]. La CP d'origine digestive présente une Médiane de survie de 6 mois toutes causes confondues et dépend de la tumeur d'origine (évolutivité et la chimio-sensibilité) [12], du fait que le pronostic de ces CP est sombre, la médiane de survie rapportée dans la littérature internationale est de 10 mois [13]. Une étude a montré que, les CP d'origine digestive, ont une survie médiane de 23.9 mois, une survie globale de 65% à 2 ans et de 13% à 5 ans [14]. La CP représente la forme dévastatrice de la progression du cancer dont le pronostic est très mauvais et dépend du primitif. Une proportion importante de patients (39.25% dans notre série) ne fait appel à la médecine moderne qu'après 6 mois, voire même 1 an, d'évolution de leur maladie. Ce retard à la consultation peut être expliqué par l'automédication et l'interprétation socioculturelle chez certains patients comme première issue.

## Conclusion

La CP a toujours été considérée comme une maladie en phase terminale. Il ressort que l'âge présente un facteur de risque essentiel dans le cas de la CP d'origine digestive, Le diagnostic de cette pathologie pose un problème stratégique, ainsi que la méconnaissance peut induire une prise en charge inadaptée. Les données présentées dans cet article peuvent ne pas être représentatives des données de la population générale de la CP, mais cependant ils donnent une idée sur l'ampleur et la gravité de cette pathologie.

### Etat des connaissances actuelle sur le sujet

- La carcinose péritonéale représente la lésion la plus fréquente du péritoine;
- Le pronostic de la carcinose péritonéale est très réservé en l'absence de prise en charge thérapeutique adaptée ou de simple traitement médical;
- La carcinose péritonéale d'origine digestive a une survie médiane de 23.9 mois, une survie globale de 65% à 2 ans et de 13% à 5 ans.

### Contribution de notre étude à la connaissance

- Les patients âgés de plus de 50 ans, sont les plus touché par la carcinose péritonéale (78.13%);
- L'âge et les antécédents familiaux présentent des facteurs de risque de l'apparition de la carcinose péritonéale d'origine digestive;
- Le retard à la consultation dépasse 6 mois chez les patients avec carcinose péritonéale d'origine digestive.

## Conflits d’intérêts

Les auteurs ne déclarent aucun conflit d'intérêts.
